# Increased Plasma Concentrations of Extracellular Vesicles Are Associated with Pro-Inflammatory and Pro-Thrombotic Characteristics of Left and Right Ventricle Mechanical Support Devices

**DOI:** 10.3390/jcdd10010021

**Published:** 2023-01-05

**Authors:** Tomasz Urbanowicz, Anna Olasińska-Wiśniewska, Kajetan Grodecki, Aleksandra Gąsecka, Krzysztof J. Filipiak, Maciej Gawlikowski, Łukasz Mucha, Marek Jemielity

**Affiliations:** 1 Cardiac Surgery and Transplantology Department, Poznan University of Medical Sciences, 61-848 Poznan, Poland; 2 1st Department of Cardiology, Medical University of Warsaw, 02-097 Warsaw, Poland; 3 Institute of Clinical Science, Maria Sklodowska-Curie Medical Academy, 00-136 Warsaw, Poland; 4 Faculty of Biomedical Engineering, Silesian University of Technology, 44-100 Gliwice, Poland; 5 F.R.K. Intra-Cordis Professor Zbigniew Religa Foundation of Cardiac Surgery Development, 41-800 Zabrze, Poland

**Keywords:** NLR 1, SIRI 2, SII 3, left ventricular assist device 4, mechanical circulatory support 5, BIVAD 6

## Abstract

Mechanical circulatory support (MCS) allows for functional left and right heart ventricle replacement. MCS induces a systemic inflammatory reaction and prothrombotic state leading to an increased risk of thrombus formation. The extracellular vesicles (EVs) are nanoparticles released from active/injured cells characterized by prothrombotic properties. Simple inflammatory parameters from whole blood count analysis have established a clinical role in everyday practice to describe immune-inflammatory activation. We hypothesized that increased plasma concentrations of EVs might be associated with the proinflammatory and pro-thrombotic characteristics of left ventricle assist device (LVAD) and right ventricle assist device (RVAD) devices. We presented a pilot study showing the concentration of peripheral blood serum, right and left ventricle mechanical assist device extracellular concentration in relation to thrombotic complication in patients treated with a biventricular pulsatile assist device (BIVAD). The observation was based on 12 replacements of pulsatile pumps during 175 days of observation. The proinflammatory characteristics of LVAD were noted. The proinflammatory and procoagulant activation by RVAD was observed. The results may provide possible explanations for the worse results of right-sided mechanical supports observed in clinical practice.

## 1. Introduction

A continuous remarkable development in mechanical circulatory support (MCS) has become an interesting alternative to heart transplantation, achieving satisfactory long-term results [1,2]. The superiority of the centrifugal pump in comparison to organ transplantation was postulated in a recent trial [3] since the centrifugal pump presents satisfactory results with a relatively low risk for complications [4].

MCS can be configurated to support the left, right, or both ventricles, giving a chance for both physiological pumps’ function replacement. Currently, the HeartMate3 (Abbott, Chicago, IL, USA) as a centrifugal pump is routinely applied to support the left heart chamber [5]. The pulsatile paracorporeal pumps, though not routinely applied, are available, including Excor (Berlin Heart Company, Berlin, Germany) [6] for pediatric patients or POLVAD (FRK IntraCordis, Zabrze, Polska) for adults [7], and allow for long-term support in contrast to results obtained from the combined two left ventricle assist devices [8].

Pulsatile pneumatic pumps presented optimal results in children [9] including pulmonary hypertension reversal and satisfactory post-transplantation survival. The comparable results between the use of axial pumps and paracorporeal pulsatiles were presented in adults [10], though the increased risk for thrombus formation required frequent (every 30 days) pumps exchange [11] and in-hospital stays are of the greatest concern.

MCS induces systemic inflammatory reaction and prothrombotic state [12] leading to an increased risk for thrombus formation [13]. Inflammatory activation may be measured by simple indexes obtained from the whole blood count, such as neutrophil to lymphocyte ratio (NLR), monocyte to lymphocyte ratio (MLR), or indexes, such as systemic inflammatory index (SII), aggregate index of systemic inflammation (AISI) and systemic inflammatory response index (SIRI) [14,15,16]. However, these indicated markers were previously evaluated and did not allow one to identify patients treated with MCS who are at high risk of thrombotic complications. Hence, new biomarkers are needed to improve risk stratification in this challenging patient population [17].

Extracellular vesicles (EVs) are heterogenous nanoparticles released from cells to body fluids, composed of cellular membrane fragments and cytoplasmic material [18]. They contain phospholipids, proteins, and non-coding fragments of ribonucleic acid (RNA) and expose surface antigens such as CD45+ [19], CD61+ [20], CD235+ [21] derived from the parent cells, which allow one to identify their cellular origin [22,23]. EVs are released by erythrocytes, endothelial cells, leucocytes, and platelets [24] as fragments of a submicron size following activation or apoptosis. EVs contribute to intercellular communication, inflammation, and coagulation processes [25,26,27,28,29,30,31,32]. The increased concentrations of plasma EVs were postulated to reflect cells’ activations and to be associated with an increased risk of cardiovascular complications, including acute myocardial infarction or stroke [33]. We hypothesized that increased plasma concentrations of EVs might be associated with the proinflammatory and pro-thrombotic characteristics of left ventricle assist device (LVAD) and right ventricle assist device (RVAD).

### The Aim of the Study

The aim of the study was to analyze plasma concentration of EVs from leukocytes (CD45+), platelets (CD61+) and erythrocytes (CD235+) in the peripheral blood, LVAD, and RVAD chambers and to evaluate EVs correlation with (i) peripheral blood inflammatory parameters and indices (C-reactive protein (CRP), procalcitonin, large unstained cells (LUC), NLR, MLR, PLR, SII, AISI, SIRI) and (ii) thrombotic debris detected in RVADs and LVADs.

## 2. Materials and Methods

The study was performed on 12 pumps (POLVAD-MED, FRK Intra-cordis, Poland) analysis in one patient awaiting heart transplantation. The 42-years-old patient with dilated cardiomyopathy, with the end-stage heart failure (class IV according to New York Heart Association Functional Classification), and a history of aortic valve replacement with mechanical prosthesis three years before, was referred for qualification to heart transplantation. The imaging examinations (echocardiography, coronary angiography and computed tomography) revealed a severely dilated left ventricle (93 mm) with reduced ejection fraction (15%), a proper function of mechanical aortic prosthesis, and no atherosclerotic changes in coronary arteries. Despite aggressive pharmacotherapy, he developed cardiac arrest and after successful resuscitation, extracorporeal membrane oxygenation (ECMO) therapy was implemented, followed by BIVAD by implantation of POLVAD after 7 days. The patient was on an urgent heart transplant list; however, he waited for an organ donor for 175 days. During the whole hospitalization, he required 12 pump exchanges due to fibrotic debris collections which were observed during daily checkups. The typical heart failure pharmacotherapy was used, as well as antiplatelet therapy (aspirin, 75 mg daily) combined with oral anticoagulation (reference international normalized ratio (INR) 2.0–3.0). The aim of the study was to analyze the relation between inflammatory markers as extracellular vesicles and the risk of clots formation. The study was approved by the local Institutional Ethics Committee (approval number 695/20).

In patients with POLVAD, the pumps’ replacement during the POLVAD function was related to visual signs of thrombus formation on paracorporeal chambers observed during daily controls [11].

During each procedure of deplantation, blood samples were taken simultaneously from both chambers and peripheral blood samples were collected and processed according to the current guidelines for studying EVs [34]. The peripheral blood and intra-pump EVS concentration were measured during each deplantation. The following day, the peripheral blood for EVs concentration was measured again to distinguish the EVs concentration related to pumps by themselves and by the clots gathering in the chambers.

### 2.1. EVs Examination

Briefly, blood samples for EVs concentration were collected in 10 mL 0.109 mol/L citrated plastic tubes (S-Monovette, Sarstedt, Hildesheim, Germany) and 4.9 mL K3-EDTA plastic probes (S-Monovette, Sarstedt, Hildesheim, Germany). Within a maximum of 15 min from blood collection, platelet-depleted plasma was prepared by double centrifugation (Centrifuge MPW-56, MPW Med. Instruments, Warsaw, Poland). The centrifugation parameters were: 2500× *g*, 15 min, 20 °C, acceleration speed 1, no brake. The first centrifugation step was done with 10 mL whole blood collection tubes. Supernatant was collected 10 mm above the buffy coat. The second centrifugation step was done with 3.5 mL plasma in 15 mL polypropylene centrifuge tubes (Greiner Bio-One B.V). Supernatant (platelet-depleted plasma) was collected 5 mm above the buffy coat, transferred into 5 mL polypropylene centrifuge tubes (Greiner Bio-One B.V., Vilvoorde, Belgium), mixed by pipetting, transferred to 1.5 mL low-protein binding Eppendorfs (Thermo Fisher Scientific, Waltham, MA, USA), and stored in −80 °C until analyzed.

Concentrations of EVs were measured by flow cytometry (A60-Micro, Apogee Flow Systems, Hertfordshire, UK). We diluted samples 2-fold to 1500-fold in in Dulbecco phosphate-buffered saline (DPBS) to achieve a count rate of less than 3000 events/s to prevent swarm detection [35]. Diluted samples were measured during 120 s at a flow rate of 3.01 μL per min. The trigger threshold was set at 14 arbitrary units of the side scatter detector, which corresponds to a side scattering cross section of 10 nm^2^. The reported concentrations describe the number of particles (a) that exceed the side scatter threshold, (b) have a diameter >200 nm as determined by Flow-SR [36], (c) have a refractive index <1.42 to exclude positively labelled chylomicrons [37], and (d) that are positive at the fluorescence detector(s) corresponding to the used label(s), per mL of platelet-depleted plasma. We measured concentrations of EVs from erythrocytes (CD235a+), leukocytes (CD45+), and platelets (CD61+). To ensure the reproducibility of our EV flow cytometry experiments, we applied the new reporting framework for the standardized reporting of EV flow cytometry experiments (MIFlowCyt-EV) [38], calibrated all detectors, determined the EV diameter and refractive index by the flow cytometry scatter ratio (Flow-SR) [36], and applied custom-built software to fully automate data calibration and processing.

### 2.2. Inflammatory Biomarkers

Peripheral blood samples were collected during each deplantation and 24 h after the procedure to assess standard inflammatory parameters and indices, which were measured with a routine hematology analyzer (Sysmex Europe GmbH, Norderstedt, Germany). C-reactive protein (CRP) and procalcitonin levels were measured by a high-sensitive enzyme-linked immunosorbent assay technique (ELISA) according to the manufacturer’s instructions. The indices, including NLR, MLR, PLR, SIRI, AISI, SII, were calculated according to the usual formulas as presented in previous reports [39].

### 2.3. Statistical Analysis

Data were tested for normality using the Shapiro–Wilk test. Continuous variables were expressed as mean (standard deviation (SD)) or median (interquartile range [IQR]), as appropriate. Categorical variables were presented as numbers (percentage). Unpaired continuous variables were compared using t-test or nonparametric Mann–Whitney test, as appropriate. Paired variables (pre- versus post-deplantation) were compared with the Wilcoxon signed-rank test. For multiple groups, one-way ANOVA was used with post hoc Bonferroni correction. The correlation between two continuous variables was measured with the bivariate Pearson correlation. All reported probability values were 2-tailed. A P value less than 0.05 was considered significant. Data were processed using the SPSS software, version 23 (IBM SPSS Statistics, New York, NY, USA).

## 3. Results

### 3.1. Extracellular Vesicles

**Figure 1:**
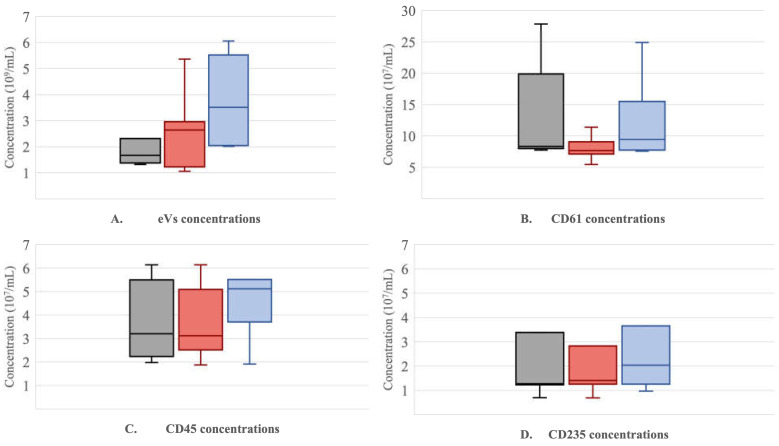
The comparison of EVs (**A**), CD61 (**B**), CD45 (**C**), CD235 (**D**) concentration in peripheral blood, left ventricle assist device (LVAD), and right ventricle assist device (RVAD). Abbreviations: LVAD—left ventricle assist device, RVAD—right ventricle assist device.

The EVs concentration in peripheral blood (1.38 (1.35–1.51) × 10^9^/mL) in LVAD (2.77 (1.25–4.24) × 10^9^/mL) and in RVAD (3.88 (2.41–4.24) × 10^9^/mL) were noted. The results of EVs-CD45 concentration were (3.2 (2.45–5.3) × 10^7^/mL vs. 3.12 (2.67–4.42) × 10^7^/mL vs. 5.12 (4.31–5.32) × 10^7^/mL in peripheral blood, LVAD, and RVAD, respectively. The EVs-CD61 peripheral blood, LVAD, and RVAD concentration was measured and presented the following concentrations: 8.35 (8.15–14.23) × 10^7^/mL vs. 7.68 (7.21–8.41) × 10^7^/mL vs. 9.41 (8.28–14.71) × 10^7^/mL, respectively. The EVs-CD235 concentrations were noticed in peripheral blood with mean values of 1.27 (1.24–2.68) × 10^7^/mL, in an LVAD concentration of 1.41 (1.31–2.79) × 10^7^/mL, and in an RVAD concentration of 2.04 (1.49–3.28) × 10^7^/mL, respectively. The graphical comparison of EVs, CD45, CD61, and CD235 concentrations in peripheral blood, LVAD, and RVAD represent Figure 1A–D.

### 3.2. Whole Blood Count and Indices Analysis

The mean values of peripheral whole blood count and inflammatory markers measured during deplantation and one day (24 h) after the procedure are presented in Table 1.

#### Pumps Analysis

**Figure 2:**
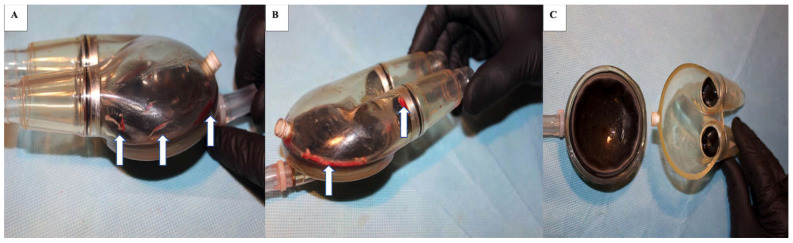
Thrombotic materials in right and left ventricle mechanical assist device. (**A**) thrombotic material (arrow) in the RVAD pump, (**B**) thrombotic material (arrow) in the LVAD pump, (**C**) no pathological debris in the mechanical assist device.

During a total of 175 days of pump function, 29 thrombi were found in the LVAD, including 6 large and 23 small thrombi located in the inflow tract (*n* = 7) and the outflow tract (*n* = 10). In the RVAD, 5 large and 14 small thrombi were detected, including 5 located in the inflow tract and 6 in the outflow tract. Figure 2A–C present the representative images of the thrombotic material. The mean (SD) time for pump function was 25 (9) days.

### 3.3. Left Ventricle Assist Device (LVAD)

The correlation between obtained data regarding EVs detected in the left chamber was performed and presented in Table 2.

**Figure 3:**
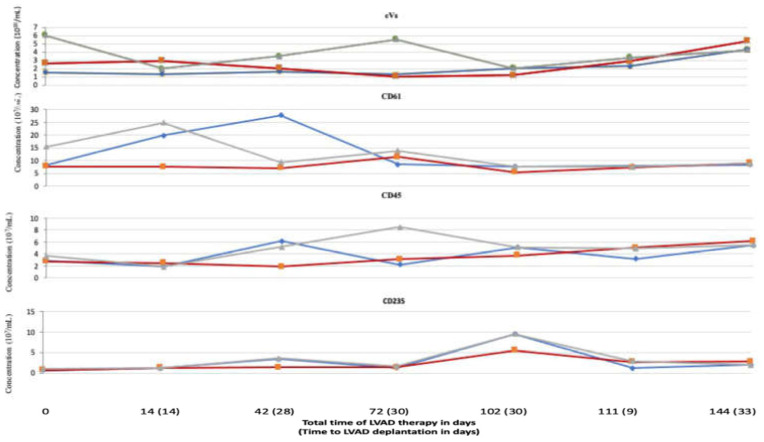
EVs, CD45, CD61, CD235 concentration in LVAD, RVAD, and peripheral blood presented in a timely manner. Abbreviations: eVs—extracellular vesicles, LVAD—left ventricle assist device. Blue line—right ventricle assist device (RVAD), red line—left ventricle assist device (LVAD), gray line—peripheral blood.

The eVs including (separate measurements of CD61, CD45, CD235) concentrations in RVAD and LVAD compared to peripheral blood eVs concentration during every system deplantation are presented in Figure 3.

### 3.4. Right Ventricle Assist Device (RVAD)

The results of correlation between EVs concentrations in the RVAD and laboratory data are presented in Table 3.

## 4. Discussion

The results of our study present the first, to our best knowledge, results of EVs concentration in mechanical circulatory support and their relation to inflammatory and thrombotic indices. Left-sided mechanical support proinflammatory characteristics were noted in our analysis as reflected by platelet EVs (CD61+) and erythrocytes EVs (CD235+) concentrations, which correlated with peripheral blood MLR. The right-sided mechanical pumps (RVAD) had proinflammatory and procoagulant activation, as reflected by leukocyte EVs (CD45+) and platelet EVs (CD61+) concentrations and peripheral blood NLR and SII. The proinflammatory and prothrombotic results in RVADs may explain the worse clinical results in RVADs [40] also after heart transplantation [41].

Extracellular vesicles (EVs) are involved in atherosclerosis, thrombosis, and inflammation. We evaluated EVs exposed from red cells (CD235+), platelets (CD61+), lymphocytes (CD45+), a total concentration of EVs above the detection threshold of our flow cytometer, and showed their proinflammatory and prothrombotic significance during biventricular mechanical support use.

Our analysis results indicate that inflammatory activation in the left-sided mechanical system is triggered by red cells and platelets. The CD235+/CD41− erythrocyte-derived antigens concentrations are related to inflammatory markers procalcitonin and MLR. The CD235+ antigens belong to red cell fragments of the submicron size that can be regarded as a prothrombotic marker [42].

The increased levels of procalcitonin and MLR were [43,44] obtained from left-sided MCS [45,46]. Procalcitonin is a marker for sepsis but has a limited sensitivity since its secretion was also observed in non-infectious states [47] and was suggested as a prognostic factor of poor prognosis in acute coronary syndromes [48]. Its increased levels were proposed, by Xiong et al., as a risk biomarker for thrombotic complications following surgery [49]. Monocyte to lymphocyte ratio (MLR) is a simple index that is mainly related to inflammatory activation, however increased thrombotic risk was also postulated [50,51].

In our study, the relation was noted between LUC and platelet CD61 in left-sided mechanical support analysis. The LUC are large unstained peroxidase negative cells that reflect to activated lymphocytes, monocytes, and lymphoblasts [52]. Their increase was observed in different clinical situations related to acute immune activation [53,54]. CD61 (platelet glycoprotein IIIa) plays a significant role in aggregation. CD61 is a protein that indicate platelets secretion due to activation secondary to the procoagulant surface presented in the Reddel et al. study [55]. The complex GP IIb-IIIa binds to fibronectin, fibrinogen, and to vitronectin and von Willebrand factor [56]. CD61 was related to microparticles released as the result more of activation than of apoptosis by Vasina et al. [57].

Right sided mechanical circulatory support stimulates pro-thrombotic action by CD61 that plays a crucial role in platelets aggregation [58]. Our finding indicates the neutrophil to lymphocyte ratio (NLR) and systemic inflammatory index (SII) as two inflammatory indices related to EVs detected in RVAD chambers. The NLR was presented as an ominous prognostic marker for inflammatory activation in previous reports [59,60,61]. It can be regarded as a novel prospective marker related to ongoing antagonism between adaptive and innate immunity [62]. The relation between RVAD and SII indicate activation of three components: neutrophils, platelets, and lymphocytes [63]. The CD45 as a marker for lymphocytes activation by signal transduction [64] was related to RVAD in our study, emphasizing the significance for inflammatory activation. CD45+ lymphocyte-derived EVs play a significant role in vascular function. They are characterized by potential paracrine function [65]. The RVAD chamber content revealed significance for EVs. Based on our results, we suggest proinflammatory and pro-thrombotic characteristics of RVAD.

Our results indicate the relation between platelets activation in RVAD expressed by CD61+ and its peripheral response by extracellular vesicles (EVs) serum concentration. EVs were revealed to transfer functional receptors as triggering platelets antigen CD61, promoting target cells proliferation and adhesion [66].

After left -sided mechanical support (LVAD) implantation, the decrease of circulating EVs was presented in follow-up irrespectively to disease etiology or gender [67], suggesting a potential improvement of endothelial function. A Roka-Moiia et al. study [68] presented the platelets dysfunction as related to EVs shear stress generation in mechanical support. Radley et al. in their review postulated leukocytes activation as a significant contribution to thrombus formation in mechanical support [69]. The proinflammatory and prothrombogenic EVs are released by leukocytes after foreign surfaces contact and shear stress. We noticed differences in erythrocytes, leucocytes, and platelet activation related to mechanical support location (RVAD vs. LVAD). Moreover, we found a relation with inflammatory activation presented by MLR, NLR, and SII indexes.

### Study Limitation

Patients with heart failure are extremely different in origin and advancement of disease, treatment regiments and pathophysiological response to pharmacotherapy. Indeed, this observation led us to evaluate changes in EVs in one particular patient, in whom the thrombotic and inflammatory milieu would not result from patient-to-patient differences but from disease and response to therapy. We avoided the risk of discrepancies between patients in terms of different hematological and inflammatory conditions in course of mechanical support. Moreover, our observations show thrombotic phenomena related to inflammatory activation which occur in BIVAD therapy and may be assigned to this population of patients.

We are, however, aware about possible limitations and difficulties in applying the conclusions of this patient study to the treatment of others. Therefore, we believe that larger studies are necessary to confirm our assumptions in different types of heart failure and transfer our results into therapeutic applications.

Despite the fact that the pulsatile pneumatic paracorporeal pumps are considered outdated due to centrifugal incorporable systems, this type of mechanical support allows satisfactory maintenance of both ventricles.

## 5. Conclusions

The proinflammatory characteristics of LVAD was presented based on CD235 as compared to peripheral blood MLR. The proinflammatory and procoagulant activation by RVAD were revealed based on CD45 and peripheral blood NLR, SII, SIRI, indicating an increased risk of thrombus formation. The results may provide the possible explanations of the worst results of right-sided mechanical supports observed in clinical practice. We are aware that these preliminary patient study results may be individual and should be confirmed in the analysis of larger population of heart failure patients.

## Figures and Tables

**Table 1:** Laboratory results obtained from the peripheral blood samples during deplantation and 24 h following the procedure.

Parameters	Deplantation	At 24 h	*p*
Whole blood count:			
WBC (median (IQR) (K/μL))	7.67 (5.55–8.18)	6.70 (5.72–7.83)	0.236
Neutrophils (median (IQR) (K/μL))	5.16 (3.10–5.67)	4.84 (3.27–5.03)	0.444
Lymphocytes (median (IQR) (K/μL))	1.34 (1.24–1.47)	1.22 (0.86–1.28)	0.026 *
Monocytes (median (IQR) (K/μL))	0.59 (0.57–0.74)	0.57 (0.46–0.66)	0.034 *
NLR (median (IQR))	3.52 (2.31–4.51)	3.89 (2.88–4.67)	0.073
MLR (median (IQR))	0.44 (0.41–0.51)	0.54 (0.36–0.65)	0.088
PLR (median (IQR))	185 (159–195)	222 (203–331)	0.003 *
SII (median (IQR))	823 (539–1118)	1014 (822–1382)	0.023 *
SIRI (median (IQR))	2.09 (1.41–2.60)	2.37 (1.62–2.85)	0.197
AISI (median (IQR))	555 (318–637)	562 (460–737)	0.728
LUC (median (IQR) (K/μL))	0.21 (0.18–0.29)	0.29 (0.17–0.40)	0.273
Hb (median (IQR) (mmol/μL))	6.2 (5.70–6.50)	6.30 (6.00–6.40)	0.533
Hct (median (IQR) (%))	30 (28–32)	31 (30–32)	0.243
MCHC (median (IQR) (mmol/L))	20.54 (20.15–20.80)	20.39 (19.92–20.58)	0.012 *
RDW (median (IQR) (%))	15.7 (14.3–18.0)	16.9 (14.4–19.1)	0.006 *
Platelets (median (IQR) (K/μL))	235 (233–265)	271 (237–285)	0.161
MPV (median (IQR) (fL))	7.4 (7.1–7.5)	7.30 (6.90–7.60)	0.497
Inflammatory markers:			
CRP (median (IQR) (mg/L))	46 (24–62)	46 (38–84)	0.754
Procalcitonin (median (IQR) (ng/mL))	0.21 (0.03–1.33)	0.86 (0.03–2.48)	0.221

Abbreviations: AISI—aggregation inflammatory systemic index, CRP—C reactive protein, Hb—hemoglobin, Hct—hematocrit, MCHC—mean corpuscular hemoglobin concentration, MLR—monocyte to lymphocyte ratio, MPV—mean platelet volume, NLR—neutrophil to lymphocyte ratio, PLR—platelets to lymphocyte ratio, RDW—red cells width, SII—systemic inflammatory index, SIRI—systemic inflammatory response index, WBC—white blood count. * statistical significance.

**Table 2:** Correlation matrix for LVAD and peripheral blood parameters and EVs.

	EVs	CD61	CD45	CD235
CRP pre	−0.541	−0.162	0.559	0.739
CRP post	−0.286	−0.714	0.214	0.857
PCT pre	−0.018	−0.216	0.775	0.937
PCT post	0.144	−0.234	0.739	0.955
Crea pre	0.750	−0.179	0.286	0.250
Crea post	0.071	−0.286	−0.214	0.286
WBC	−0.571	−0.321	−0.179	0.464
Neutrophils	−0.643	−0.250	0.179	0.679
Lymphocytes	−0.071	−0.536	−0.179	0.464
Monocytes	−0.432	0.378	−0.703	−0.595
NLR	−0.536	0.107	0.357	0.607
MLR	−0.393	0.536	−0.571	−0.821
PLR	−0.036	0.750	0.286	−0.393
SII	−0.536	0.107	0.357	0.607
SIRI	−0.714	0.036	0.036	0.393
AISI	−0.750	0.107	−0.071	0.250
LUC	−0.071	−0.857	0.143	0.714
Rbc	0	0.536	0.393	0.250
Hb	0.523	0.505	0.126	−0.180
Hct	0.509	0.473	0.036	−0.255
MCHC	−0.214	−0.107	−0.179	−0.107
RDW	−0.072	−0.270	0.775	0.847
Platelets	−0.393	0.536	0.286	0.179
MPV	0.324	0.144	0.901	0.541

Abbreviations: AISI—aggregation inflammatory systemic index, crea—serum creatinine, CRP—C reactive protein, Hb—hemoglobin, Hct—hematocrit, LUC—large unstained cells, MCHC—mean corpuscular hemoglobin concentration, MLR—monocyte to lymphocyte ratio, MPV—mean platelet volume, NLR—neutrophil to lymphocyte ration, PCT—procalcitonin, PLR—platelets to lymphocyte ratio, post—24 h after deplantation, pre—before deplantation, RDW—red cells width, SII—systemic inflammatory index, SIRI—systemic inflammatory response index, WBC—white blood count.

**Table 3:** Correlation matrix for RVAD blood biomarkers and microparticles.

	EVs	CD61	CD45	CD235
CRP pre	−0.108	−0.703	0.595	0.667
CRP post	−0.429	−0.786	0.357	0.987
PCT pre	−0.342	−0.775	0.432	0.685
PCT post	−0.144	−0.847	0.523	0.739
Crea pre	−0.214	−0.429	−0.179	0.179
Crea post	0.071	0	0.250	0.357
WBC	−0.143	−0.107	0.536	0.607
Neutrophils	−0.143	−0.464	0.714	0.750
Lymphoctes	−0.321	−0.071	0.107	0.536
Monocytes	0.505	0.667	0.324	−0.342
NLR	0.036	−0.393	0.857	0.536
MLR	0.607	0.643	0.107	−0.607
PLR	0.571	0.071	0.214	−0.536
SII	0.036	−0.393	0.857	0.536
SIRI	0.286	−0.321	0.893	0.536
AISI	0.286	0	0.750	0.321
LUC	−0.357	−0.714	0	0.857
Rbc	0.179	0.143	0.536	−0.107
Hb	0.108	0.523	−0.090	−0.541
Hct	0.036	0.600	−0.182	−0.582
MCHC	−0.250	0.429	−0.321	−0.179
RDW	−0.216	−0.955	0.378	0.703
Platelets	0.536	0.071	0.750	−0.036
MPV	0.306	−0.685	0.252	0.180

Abbreviations: AISI—aggregation inflammatory systemic index, crea—serum creatinine, CRP—C reactive protein, Hb—hemoglobin, Hct—hematocrit, LUC- large unstained cells, MCHC—mean corpuscular hemoglobin concentration, MLR—monocyte to lymphocyte ratio, MPV—mean platelet volume, NLR—neutrophil to lymphocyte ration, PCT—procalcitonin, PLR—platelets to lymphocyte ratio, post—24 h after deplantation, pre—before deplantation, RDW—red cells width, SII—systemic inflammatory index, SIRI—systemic inflammatory response index, WBC—white blood count.

## Data Availability

Data will be available for 3 years following publication via contacting the corresponding author (tomasz.urbanowicz@skpp.edu.pl) after reasonable requirements.
